# Supplementary material for: A supply and demand intervention increased fish consumption among rural women: A randomized, controlled trial
Source: PLoS One. 2026 Feb 19;21(2):e0340861. doi: 10.1371/journal.pone.0340861 (PMC12919792; doi:10.1371/journal.pone.0340861)
Supplement: S1 Table — (DOCX) [file pone.0340861.s001.docx]

**Table S1**. List of species groupings used for catch monitoring in the Peskas digital monitoring system.

| code | catch_name | Lowest_tax_id | isscaap_taxcode | interagency_code | Lowest tax category |
| --- | --- | --- | --- | --- | --- |
| 0 | Zero catch | - | - | - | - |
| 1 | Short bodied mackerel | [*Rastrelliger*](http://researcharchive.calacademy.org/research/ichthyology/catalog/fishcatget.asp?genid=5469)*[brachysoma](http://researcharchive.calacademy.org/research/ichthyology/catalog/fishcatget.asp?spid=21251%22%20\\t%20%22_blank%22%20\\o%20%22Catalog%20of%20Fishes%20-%20Species)* | 17501014XX | RAX | Species |
| 2 | Mackerel scad | *Decapterus macarellus* | 17023043XX | SDX | Species |
| 3 | Jacks/Trevally/Other Scad | Carangidae | 17023XXXXX | CGX | Family |
| 4 | Tuna/Bonito/Other Mackerel | Scombridae | 17501XXXXX043 | TUN | Family |
| 5 | Fusilier | Caesionidae | 17000XXXXX | CJX | Family |
| 6 | Sardines/pilchards | Clupeidae | 12105XXXXX | CLP | Family |
| 7 | Garfish | Hemiramphidae | 1470300315 | GZP | Family |
| 8 | Soldierfish | Holocentridae | 16111006XX | YDX | Family |
| 9 | Long tom | Belonidae | 14701XXXXX | BEN | Family |
| 10 | Snapper/seaperch | Lutjanidae | 17032027XX | SNA | Family |
| 11 | Spinefoot | Siganidae | 17407001XX | SPI | Family |
| 12 | Grouper | Serranidae | 17002042XX | GPX | Family |
| 13 | Emperor | Lethrinidae | 17038XXXXX | EMP | Family |
| 14 | Surgeonfish | Acanthuridae | 17402XXXXX | SUR | Family |
| 15 | Parrotfish | Scaridae | 17065XXXXX | PWT | Family |
| 16 | Triggerfish | Balistidae | 19010XXXXX | TRI | Family |
| 17 | Flying fish | Exocoetidae | 14704XXXXX | FLY | Family |
| 18 | Unicornfish | Acanthuridae | 17402XXXXX | SUR | Family |
| 19 | Mojarra/Silverbelly | Gerreidae | 17046036XX | MOJ | Family |
| 20 | Jobfish | Lutjanidae | 17032217XX | LWX | Family |
| 21 | Wrasse | Labridae | 17063XXXXX | WRA | Family |
| 22 | Barracuda | Sphyraenidae | 17710001XX | BAR | Family |
| 23 | Chub | Kyphosidae | 17047XXXXX | KYX | Family |
| 24 | Octopus | Octopodidae | 32109005XX | OCZ | Family |
| 25 | Moonfish | Menidae | 1702632701 | MOO | Family |
| 26 | Moontail bullseye | Priacanthidae | 1701102606 | BWH | Family |
| 28 | Ponyfish | Leiognathidae | 1703516906 | LGE | Family |
| 29 | Bream | Nemipteridae | 17033230XX | MOB | Family |
| 30 | Blackspot sweeper | Pempheridae | 1704800202 | MHL | Family |
| 31 | Goatfish | Mullidae | 17041251XX | GOX | Family |
| 32 | Mullet | Mugilidae | 16501XXXXX | MUL | Family |
| 33 | Terapon | Terapontidae | 17004089XX | THO | Family |
| 34 | Shark | Carcharhinidae | 199XXXXXXX053 | SKH | Family |
| 35 | Sweetlips | Haemulidae | 17036XXXXX | GRX | Family |
| 36 | Moray | Muraenidae | 14306XXXXX | MUI | Family |
| 37 | Sergeant | Pomacentridae | 17062XXXXX | DSF | Family |
| 38 | Shrimp | Caridae | 22801XXXXX | PEZ | Infraorder |
| 39 | Butterflyfish | Chaetodontidae | 17052116XX | IHX | Family |
| 40 | Cardinalfish | Apogonidae | 17012XXXXX | APO | Family |
| 41 | Cuttlefish | Sepiidae | 32102002XX | IAX | Family |
| 42 | Javelin/Grunt | Haemulidae | 17036209XX | BGX | Family |
| 43 | Sailfish | Istiophorus | 1750300402 | SFA | Genus |
| 44 | Cobia | Rachycentridae | 1702222101 | CBA | Family |
| 45 | Crab | Brachyura | 231XXXXXXX | CRA | Infraorder |
| 46 | Dolphinfish | Coryphaenidae | 17028XXXXX | DOX | Family |
| 47 | Bannerfish | Chaetodontidae | 17052116XX | IHX | Family |
| 48 | Milkfish | Chanidae | 1220200101 | MIL | Family |
| 49 | Threadfin | Polynemidae | 17077XXXXX | THF | Family |
| 50 | Cockles | Cardiidae | 31623XXXXX | COZ | Family |
| 51 | Remora | Echeneidae | 17042XXXXX | ECN | Family |
| 52 | Tripodfish | Triacanthidae | 19002XXXXX | PUX | Family |
| 53 | Wolf herring | Chirocentridae | 12111002XX | DOS | Family |
| 54 | Stingrays | Myliobatiformes | 110XXXXXXX | SRX | suborder |
| 55 | Sicklefish | Drepaneidae | 17050132XX | DRZ | Family |
| 56 | Lobster | Nephropidae | 22901001XX | SLV | Family |
| 58 | Seaweed | Archaeplastida | 799XXXXXXX005 | SWX | Kingdom |
| 57 | Sea cucumber | Holothuridae | 694XXXXXXX | CUX | Class |
| 300 | Other |  | 199XXXXXXX010 | MZZ |  |
| 999 | Unknown |  | 199XXXXXXX010 | MZZ |  |
