# Supplementary material for: A supply and demand intervention increased fish consumption among rural women: A randomized, controlled trial
Source: PLoS One. 2026 Feb 19;21(2):e0340861. doi: 10.1371/journal.pone.0340861 (PMC12919792; doi:10.1371/journal.pone.0340861)
Supplement: S2 Table — (DOCX) [file pone.0340861.s002.docx]

**Table S2.** Differences in household fish purchasing patterns in rural, inland Timor-Leste at endline, comparing the control group as either districts that did not receive a FAD, or a district where a FAD was placed, and catch did not increase, to the district where the FAD was placed, and catch increased.

| **Treatment arm** | **N (households)** | **Proportion of households purchasing fish in previous 7 days** | **Prevalence ratio (95% CI)*** | **Mean quantity in grams (CI) of fish purchase/household member/week**** | **Back-transformed β (95% CI)***** |
| --- | --- | --- | --- | --- | --- |
| Control (all districts with data except Bobonaro) | 375 | 60% | Ref | 58.7 (45.8, 75.2) | Ref |
| FAD (Bobonaro) | 92 | 75% | 2.1 (0.9, 4.9) | 95.9 (58.2, 157.9) | 1.20 (0.49, 2.97) |

*Differences in prevalence were assessed by treatment arm using a logistic regression model, controlling for season, household wealth, and fish purchase at baseline (Y/N) with robust standard errors controlling for clustering at the village level 
**Geometric means and CI back transformed from logged values presented 
***Mean differences analyzed by log-transformed values assessed using a generalized linear model, controlling for household fish purchases at baseline and household wealth with robust standard errors controlling for clustering at the village level
