# Supplementary material for: A supply and demand intervention increased fish consumption among rural women: A randomized, controlled trial
Source: PLoS One. 2026 Feb 19;21(2):e0340861. doi: 10.1371/journal.pone.0340861 (PMC12919792; doi:10.1371/journal.pone.0340861)
Supplement: S3 Table — (DOCX) [file pone.0340861.s003.docx]

**Table S3.** Fish consumption by women in the previous 24 hours at endline, comparing the control group as either districts that did not receive a FAD, or a district where a FAD was placed, and catch did not increase, to the district where the FAD was placed, and catch increased.

| **Treatment arm** | **N (women total)** | **Women consuming fish in the previous 24 hours** | **Prevalence ratio (95% CI)** |
| --- | --- | --- | --- |
| Control (all districts with data except Bobonaro) | 244 | 8% (n=19) | REF |
| FAD (Bononaro) | 67 | 25% (n=17)** | 8.5 (1.7, 41.6) |

*Differences in prevalence were assessed by treatment arm using a logistic regression model, controlling for household wealth with robust standard errors controlling for clustering at the village level **p<0.05
