# Supplementary material for: A supply and demand intervention increased fish consumption among rural women: A randomized, controlled trial
Source: PLoS One. 2026 Feb 19;21(2):e0340861. doi: 10.1371/journal.pone.0340861 (PMC12919792; doi:10.1371/journal.pone.0340861)
Supplement: S1 File — (PDF) [file pone.0340861.s004.pdf]

VIII GOVERNO CONSTITUCIONAL
MINISTERIO DA SAÚDE
INSTITUTO NASIONAL DE SAÚDE
Gabinete Directora Executivo

HUMAN RESEARCH ETHICS COMMITTEE

Application Form

1. Principal Investigator:

| | |
|---|---|
| a. Name | : Alex Tilley |
| b. Institutions | : WorldFish |
| c. Office Address | : Jalan Batu Muang, Bayan Lepas, Penang, Malaysia |
| d. Telephone | : +601139021439 |
| e. Fax | : |
| f. Email | : a.tilley@cgiar.org |

**2. Research title** (Assessing adherence to standard precautions and risk taking behaviors amongst health care professionals)

| |
|---|
| The effects of fish aggregating devices and nutrition SBCC on fish consumption in Timor-Leste: A randomized controlled trial |

3. Simplified Project Title (Optional)

| |
|---|
| FADs and fish consumption in Timor-Leste |

4. Organization Accepting Responsibility for Project

a. CHRD MoH ☐
b. UNTL ☐
c. Others (Please Identify) : …MAP (DGP)……………………………………………… ☐

5. Enrolment:

a. Students Only :Name of Course :PhD/ Master Degree / Under Degree ☐
b. Researchers ☐

PART A. THE INVESTIGATOR

| | |
|---|---|
| Principal Investigator's Name: | Alex Tilley PhD |
| Qualifications: (Minim bachelor or strata 1 (S1) with experience of research) | a. Strata 1 (S1)<br>b. Strata 2 (S2)<br>c. Strata 3 (S3) |

| | |
|---|---|
| | d PhD….. **X** |
| Organization Affiliation: | WorldFish (ICLARM) |
| Position: | Scientist |
| Address | Ministerio de Agrikultura no Peska, Av. Presidente Nicolau Lobato, Comoro, No. 5, Dili |
| Phone Number:<br>Mobile:<br>Fax Number: | +601139021439 (Malaysia)<br>+67077734633 (Timor-Leste) |
| Email: | a.tilley@cgiar.org |
| Summary of expertise relevant to this research | Dr. Tilley has been working for WorldFish in partnership with the MAP in Timor-Leste since early 2016, developing and leading research in small-scale fisheries and designing outreach and communications related to improved livelihoods and nutrition from fish and fisheries. Dr. Tilley has published internationally on Timor-Leste fisheries, livelihoods, technologies and nutrition and designed and established the Timor-Leste National fisheries monitoring system, PeskAAS. |
| Please declare any competing interests | None |

| | |
|---|---|
| Co-Investigator's Name: | Dedisio Jose Lay Ximenes |
| Qualifications: | Bachelor of Agriculture, Masters of Science in Agriculture (Soil Science) |
| Organization Affiliation: | Mercy Corps |
| Position: | MERL Manager |
| Address | Mercy Corps Timor-Leste, Palm Business Center, Hudi Laran, Dili, Timor-Leste |
| Phone Number:<br>Mobile:<br>Fax Number: | Office: +670 33 12 726<br>Mobile: +670 7755 0722<br>Fax: N/A |
| Email: | dximenes@mercycorps.org |
| Summary of expertise relevant to this research | Dedisio is the Manager of Mercy Corps' Monitoring, Evaluation, Research and Learning (MERL) team. He has 12 years' experience working on development initiatives in Timor-Leste, including 5 years leading MERL activities. As the MERL Manager, he provides leadership for all aspects of MERL within Mercy Corps Timor-Leste, including oversight of data management systems, from collection to analysis and use. This includes oversight of data cleaning, storage and protection protocols. As a research lead, he has supported several national and program level assessments. This includes prior work on rural data collection, enumerator training and management of survey implementation. Additionally, Dedisio Ximenes has served as a research lead |

| | |
|---|---|
| | and focal person for support to Ministry of Agriculture on their Rapid Food Security Assessment. He has prior experience supporting INS approvals through his leadership on surveys including the Cost of Diet study for HATUTAN program. |
| Please declare any competing interests | None |
| | |
| Co-investigator's Name: | Mario Pereira |
| Qualifications: | Bachelor of Applied Sciences Majoring in Aquaculture, and Fisheries Management |
| Organization affiliation: | WorldFish |
| Address: | WorldFish, MAP, Av. Presidente Nicolau Lobato, Comoro, Dili |
| Phone Number<br>Mobile Number<br>Fax Number<br>Email | a. ………………………<br>b. +670 77851358<br>c. ………………………………………<br>d. m.pereira@cgiar.org |
| Summary of expertise relevant to this research | Mario is the country representative of WorldFish and has 15 years' experience serving as a technical advisor on fisheries and livelihoods projects for FAO and WorldFish. Mario will be responsible for in-country coordination of the study and acts as the primary liaison with GOTL partners the Ministry of Agriculture and Fisheries. |
| Please declare any competing interests | None |

PART B. THE PROJECT

1. **Type of project:** (Formatting Tip: Tick all relevant boxes by double-clicking on the box and marking 'Default Value' as 'Checked'.

| | |
|---|---|
| x | Funded |
| | Un-funded research |
| | Audit |
| | Clinical trial notification scheme (*Attach Evidence of Insurance) |
| | Staff research |
| | Student – Master Candidate research |
| | Student – PhD Candidate |

2. Is this project a continuation of a current or previous project with ethics approval?

   a. Yes ☐
   b. No ☒ x

IF YES, please provide HREC identification number: ……………………………………..……….

3. Has this project been submitted to any other ethics committee?

a. Yes b. No ☐

IF YES, please provide the following details:

☒ x

| Ethics Committee (Include Human, Animal and Biosafety Committee) | Status (To be Submitted, Approved, Not approved) | Date | Copy of Ethics Approval Attached |
|---|---|---|---|
| | | | |

4. Proposed commencement date of project: 1 October 2020…..…………………..……..……..
5. Proposed completion date of project: 31 October 2022………

6. **Summary of the project**:(In the box below, describe the project in 100 words or less)

This study aims to test and compare the effects of two treatments on the rate and volume of fish consumption in upland areas as a proxy for improved dietary diversity and micronutrient intake: Treatment 1 is the installation of nearshore, moored fish aggregating devices (FADs) to improve catch rates. Treatment 2 is the development and distribution of social and behaviour change messaging (SBCC) on the dietary benefits of consuming fish. Our study is designed to explore how improved supply of fish, and the provision of information, influence the frequency decision to purchase fish.

7. **Background to the project:** (Briefly describe the history of the topic you intend to address. A formal literature review should have been conducted and be evident in your application)

Timor-Leste is a country afflicted by multiple burdens of malnutrition. At 49.8% (43.4–56.2%), It has one of the world's highest rates of stunting (low height for age) in children (Kinyoki et al., 2020). Stunted growth is multifactorial; in Timor-Leste, a contributing cause is likely to be a monotonous diet (Provo et al., 2017). There is mounting evidence that fish is an underutilised resource that has the potential to increase dietary quality, but consumption of fish is low at ~6kg/person/yr (AMSAT International, 2011) compared to neighbouring country (Indonesia ~100kg/person/yr) and global averages (~20kg/person/yr). However, this 6kg average figure masks the large variation in fish consumption; coastal communities consume ~17 kg/person/year, while inland communities consume fish only seasonally, resulting in a consumption rate of ~4kg/person/yr (AMSAT International, 2011).
Fish aggregating devices (FADs) are a technology to concentrate fish to make them easier to find and catch, and there is evidence of their improving catch rates in both inshore (Tilley et al., 2019) and offshore settings (Albert *et al.*, 2014; Sharp, 2014; Tilley *et al.*, 2019). Despite being commonly and contentiously used in high-value oceanic fisheries, anchored nearshore FADs are also utilized by small-scale fishers to catch small pelagic fishes such as mackerels and scads.
Fish is highly sought-after animal-source food in Timor-Leste, yet given the low catch volumes landed by the small national fleet of mostly non-motorized canoes, and limited road and market infrastructures inland, the flow of fish inland is constrained (Lopez-Angarita et al. 2019). FADs provide access to a more abundant and potentially sustainable stock of fish than traditionally targeted reef fisheries in Timor-Leste. Investigating the barriers for fish from small-scale fishers to reach the populations in need is important for understanding the contribution of fisheries to nutrition security.
Investigating the barriers for fish from small-scale fishers to reach the populations in need is important for understanding the contribution of fisheries to nutrition security. Proximity to fisheries is associated with an increase in fish consumption in children in Sub-Saharan Africa (O'Meara et al, in preparation); however, more information is needed to determine if this relation holds in other contexts. Fish aggregating devices can provide access to a more abundant and potentially sustainable stock of fish than traditionally targeted reef fisheries in Timor-Leste. Promoting a healthy diet and optimal complementary feeding practices through contextualized SBCC is another avenue to improve dietary quality and eventual health outcomes. The influence of social and behaviour change communication (SBCC) on diets and expenditures is mixed (Byrd *et al.*, 2019; Lindsey, 2017), but some programs have

| |
|---|
| had success (Menon *et al.*, 2020). Thus, raising awareness about the importance of fish to a healthy diet may lead to increased consumption patterns. |

8. **Aims of the project:** (Describe your primary research question in 100 words or less:

| |
|---|
| The overall purpose of this research program is to improve knowledge of the economic, social and cultural drivers of fish consumption in rural areas of Timor-Leste. |

9. Nature of Research
This research project can be described as (tick ✓ all that apply):

| | |
|---|---|
| ☐ | Behavioural observation |
| ☐ | Self-report questionnaire<br>(please attach a copy to this application) |
| X | Interview<br>(please attach a copy of the interview content to this application) |
| ☐ | Qualitative methodology e.g. focus groups |
| ☐ | Psychological experiments |
| ☐ | Epidemiological studies |
| ☐ | Data linkage studies |
| ☐ | Psychiatric or clinical psychology studies |
| ☐ | Human physiological investigation |
| ☐ | Biomechanical device(s) |
| ☐ | Human tissue |
| X | Other (specify below) Fisheries monitoring |

10. Type of participants

| | |
|---|---|
| ☐ | Prisoners |
| ☐ | Refugees |
| ☐ | Internally displaced persons |
| ☐ | Pregnant women |
| ☐ | Members of the armed services |
| ☐ | Mentally ill |
| ☐ | Intellectually impaired |
| ☐ | Unconscious or critically ill patients |
| ☐ | Others (…………………………………) |

| | |
|---|---|
| | |

If you ticked 'YES' to any of the above, please provide details

| |
|---|
| Some of the participants we interview may be pregnant, as we want to understand the role of fish in pregnant women's diets, as well as lactating mothers, and mothers of children under 5 years old. |

11. Are you researching patients (i.e., subjects receiving health care)?

[ ] Yes
[X] No

If YES, list the procedures/techniques which would not form part of routine clinical management.

| |
|---|
| |

12. What is the sample size for the study? How will this sample size allow the aims of the study to be achieved? Note: Investigators must be satisfied that the potential benefits of the research justify any discomforts or risks towards the participants. When research is potentially harmful, the number of participants should be minimized, although not to the extent that the scientific value of the research is undermined.

| |
|---|
| This study will take place across 6 districts (Dili, Liquica, Manatuto, Manufahi, Bobonaro, Covalima). We will interview the woman head of 720 households in 24 villages across these municipalities (~30 HH per village). |

13. How will the participants be recruited?

Note: Researchers must avoid real or perceived coercion in their initial contact with participants

| |
|---|
| The participants will be recruited through already established Village Savings and Loan Associations (VSLAs) set up by project partner Mercy Corps. Any additional households interviewed to make up the sample size will be selected at random from within the same communities. |

14. Does recruitment involve a direct personal approach from the researchers to the potential participants?

[ ] Yes
[x] No

If YES, explain how the real or perceived coercion from researchers for potential participants to enrol has been addressed

| |
|---|
| |

15. Will participants receive any reimbursement of out-of-pocket expenses, or financial or other 'rewards' as a result of participation?

[ ] Yes
[x] No

If YES, what are the amount or nature of the reward and the justification for this?

| |
|---|
| |

Note. Volunteers may be paid for the inconvenience or reimbursed for time spent or transport costs. However, payment must not be so large as to be an inducement to participate.

16. Is the research targeting any particular ethnic or community group?

[X] **Yes**

No

If YES, which group is being targeted?

| |
|---|
| The participants will be recruited through already established Village Savings and Loan Associations (VSLAs) set up by project partner Mercy Corps. Participation in these groups is voluntary. Any additional households interviewed to make up the sample size will be selected at random from within the same communities. |

Note. As far as possible, there should be no discrimination on the basis of sex, age or race beyond that inherent in the research. Investigators must ensure that there are no unintended or unconscious biases in the sampling which might represent an unfair imposition on particular groups of people.

17. Will it be used for research purposes only or will it be later included in reports or publications?

| |
|---|
| This research will be analyzed and written up for publication in policy briefs for GOTL, project reports for GOTL and donors, and peer-reviewed scientific journals. |

18. How will consent be obtained? (Attach consent form if appropriate)

| |
|---|
| We will obtain written consent (by collecting signatures on a digital consent form prior to the survey questionnaire). Please see the attached survey form – highlighted Section B. |

19. How will the results of the study be disseminated (e.g., publication in a journal, presentation at scientific meetings, etc)

| |
|---|
| This research will be analysed and written up for publication in policy briefs for GOTL, project reports for GOTL and donors, and peer-reviewed scientific journals. Additionally, results will be shared at local and national meetings with stakeholders in Timor-Leste, at scientific meetings, and through local and international social media campaigns. |

20. How will feedback be made available to participants?

| |
|---|
| Following the end line survey of the project, participants will be presented the results of the trial during their VSLA meetings in their village. Furthermore, they will be able to request further information on any element of the project, so long as it does not affect personal privacy of participants. |

21. How will the confidentiality of the data, including the identity of participants, be ensured during collection and dissemination? (e.g., by coding or de-identification)

| |
|---|
| Trial participants and survey respondents will be anonymized through coding of the responses. The individual identifiers will be held by the PI and investigators only for analytical needs, and will not be disseminated. Respondent household locations will be aggregated to community level in any maps or figures. |

22. Is there any possibility that information of a personal nature could be revealed to persons not directly connected with this research?

| YES (Please give details): | NO<br>X |
|---|---|

23. What is the proposed storage location of, and access to, materials collected during the study?

Note. In general, the data and materials collected in a research study should be held in a secure location on the institution's premises.

| |
|---|
| Data will be collected on digital tablets and uploaded to a central database immediately upon connection to a viable network following completion of the survey. Once the survey has been verified as complete and accurate, the local version is deleted remotely from the tablet. All centrally stored data will be password protected. |

24. How long will materials collected during the study (including files, audiotapes, questionnaires, videotapes, photographs) be retained for after the study, and how will they ultimately be disposed of?

Aggregated, anonymized survey data will be published open access following the completion of all project activities and publications. No physical materials of a personal nature will be collected during the study.

25. Describe details of the research and all the risks involved. Indicate the rate at which these risks are expected to occur. Indicate what facilities and trained personnel are available to deal with such psychological or physical or problems.

The project involves interviews with household members. There are no personal health risks associated with this sampling. The questions relate to diet diversity and frequency, with some questions on household income and expenditure, and are very unlikely to cause any psychological stress. The rural and remote location of some sample villages presents some travel risk requiring 4x4 vehicles.

26. Abstract of the Research Proposal (short description of background, research problems, data collection techniques, time and location, and data or information that will be explored)

Timor-Leste is one of the world's most malnourished nations where micronutrient-deficient diets are a contributing factor to the prevalence of child stunting of 49.8%. Fish are an important source of micronutrients and one that may assist the country's predominantly rural population of agriculturalists to exit poverty and malnutrition. However, a small national fishing fleet producing low catch volumes place fish out of reach of most inland and upland populations where it is needed most. Fish consumption is very low in rural areas compared to coastal, regional and global averages. This trial is a one-year, cluster-randomized, partially masked controlled trial among families living in rural, inland Timor-Leste. This trial aims to test and compare the effects of two treatments, alone and in combination, on the frequency and volume of fish consumption in upland areas as a proxy for improved dietary diversity and micronutrient intake. Treatment 1 is the installation of nearshore, moored fish aggregating devices (FADs) to improve catch rates with existing fishing gears. Treatment 2 is the distribution of social and behaviour change communication (SBCC) on the dietary benefits of consuming fish. Villages in inland communities will be randomized to receive treatment 1, treatment 2, both treatments, or neither treatments. Households with one child under five will be recruited, and data will be collected at baseline (prior to the rollout of the treatments) and end line. Our study will determine the impact of an improved supply of fish, along with nutrition-oriented SBCC messages, on the fish purchasing and consumption practices of rural, inland households. Findings such as these are urgently needed by small island developing states in order to make policy and investment decisions on how best to improve households' diets using locally-available, nutrient-dense foods such as fish. Investments such as these are needed to break the cycle of malnutrition.

27. Advantages of the Research (identify stakeholders and how they hope to use the research findings)

This research is aligned specifically with the National Development Strategy of Timor-Leste in two key areas: 1) to reduce malnutrition, specifically in inland areas by increasing dietary diversity and micronutrient availability, and 2) to increase the production and contribution of fish and fisheries to the agriculture sector as a key source of animal protein. As such, the stakeholders of this study include the Timorese people the fisheries and development officers of MAF and the GOTL as they will be partners and collaborative recipients in testing approaches to achieve these goals.

28. Methodology of Research

Description of (a) Conceptual framework, (b) Time and location of research, (c) Type of research / research design, (d) Variables and operational definitions, (e) Instruments and data collection techniques

**Objectives and hypothesis**

Our trial evaluates the effectiveness of increased fish production and social and behaviour change communication (SBCC) on the volume and frequency of fish consumption in rural households in Timor-Leste. Findings from our study will inform governments, practitioners, and donors on the implementation of effective interventions to improve household diets through increased fish consumption.

At the highest level, we hypothesize that communities exposed to increased fish production and SBCC will result in a significantly larger positive change in fish consumption volume and frequency compared to only-SBCC and only FAD treatment arms and the control households.

**Trial design**

Our study will implement a one year-long, cluster randomised controlled trial (RCT) among rural inland households. We will test the effects of a bundled intervention addressing both supply-side and demand-side constraints to consumption of fish.

On the supply side, the experiment will involve the deployment of two fish aggregating devices in the nearshore fishing grounds of a coastal fishing village with already established trading links to inland study sites. Randomisation of the interventions will occur at the site level. Using WorldFish and government data on fisheries landing sites in six municipalities, rural coastal fishing villages with over 10 active fishing vessels will be listed. Villages that already have or utilise FADs will be excluded. Six villages (one per municipality) will then be randomly assigned to one of two RCT arms, namely FAD (4 villages) and non-FAD (2 villages) (Figure 1). Inland communities linked to each of the coastal villages will then be identified. The coastal villages are geographically dispersed, hence minimizing the risk of contamination. The efficacy of nearshore FADs at increasing catch rates of fish has already been tested in a prior study (Tilley et al., 2019)

On the demand side, 3-6 inland communities within a 30 km radius of each coastal village that has an established Village Savings and Loans Associations (VSLA) by Mercy Corps will be randomised into SBCC and non-SBCC. A further inclusion criterion for the villages is that fish traders from the coastal landing sites confirm through interview that they currently sell fish products to those respective villages. Villages will be randomly allocated to treatment or control arms using an Excel random number table to a 1:1 allocation ratio. The principal researcher will generate the allocation sequence.

Within each SBCC inland community, we will partner with Mercy Corps to implement a campaign to provide nutrition knowledge/messages through Village Savings and Loans Associations (VSLA). The VSLA members will receive the core messaging about fish nutrition as part of facilitated discussions, interactive learning sessions, and a video-based facilitated dialogue. At community level (N = 12), Mercy Corps will promote increased fish consumption through mass attendance events through marketing of fish vendors, interactive learning, and *edu-tainment* competitions promoting fish consumption. The non-SBCC communities will not receive nutrition campaign messages. SBCC will raise awareness of the benefits of eating fish as part of a balanced diet, with a particular focus on pregnant and lactating mothers, and children under 5 years of age.

At both levels, sites will be randomly allocated to treatment or control arms using an Excel random number table to a 1:1 allocation ratio. The principal researcher will generate the allocation sequence.

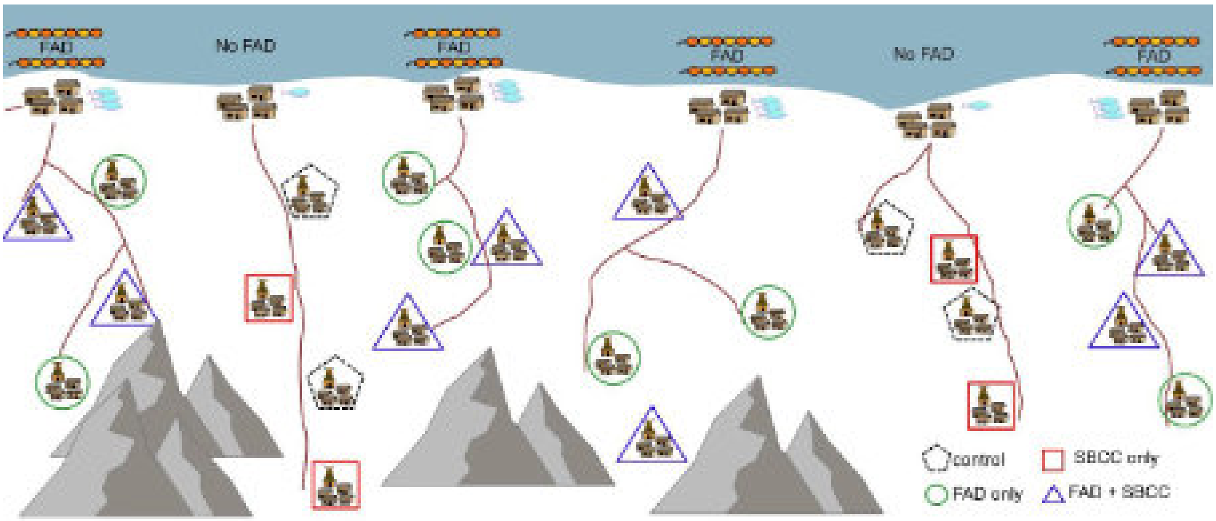


***Figure 1****. A sketch representation of the two treatment levels of the randomised controlled trial. 1. Coastal nearshore fish aggregating devices and 2. Social and behaviour change communication in rural inland communities in Timor-Leste. This diagram is a visualization only and does not represent the location of villages in the study.*

Research team

This study will be undertaken by WorldFish Timor-Leste staff, WorldFish scientists across different research programs, and in partnership with Mercy Corps.

WorldFish will lead the design and implementation of the study on the ground.

Mercy Corps are responsible for developing the SBCC messaging tools in collaboration with WorldFish nutrition scientists. The study will also leverage the Mercy Corps network of Savings and Loans Associations (VSLA).

Four enumerators will conduct the baseline, mid line and end line surveys. Each survey will take ~9 weeks to complete, based on each enumerator working 5 days per week conducting 4 surveys per day. At this rate, it will take approximately 2 days to complete each village.

Study site(s)

This study will be carried out in six coastal districts of Timor-Leste (Dili, Liquica, Bobonaro, Manatuto, Manufahi and Covalima)(Figure 2). These municipalities were chosen based on the coverage of Mercy Corps programming of VSLA groups. North and south coast municipalities were selected to broaden the applicability of the outcomes to national level programming, due to the bathymetric and ecological differences between the coasts (Lopez-Angarita et al., 2019). Six coastal villages will be randomly assigned to FAD treatment (N=4) or FAD control groups (N=2). The network of inland community markets supplied by traders from these six coastal fishing communities, and with a pre-established Mercy Corps VSLA, will be mapped prior to randomized selection. These VSLA communities will be listed and randomly assigned into SBCC (treatment) and non-SBCC (control) groups with a total of 12 villages under treatment and 12 control villages.

An inclusion criterion for the villages is that established and current fish traders from the coastal landing sites confirm through interview that they are supplying the upland village with fish products from coastal landing sites.

| **Municipality** | **Coastal sites**[1] | **Upland aldeias** | |
|---|---|---|---|
| **Bobonaro (97,762)** | Atabae (10,963) | Irlelo - | Fatubesi – 418 |
| | | Lebos – 966 | Liabote – 600 |
| | | Rairobo – 1,632 | |
| **Manufahi (53,691)** | Betano (30,673) | Loti – 657 | Nonobuco |
| | | Raimera - 976 | |
| **Liquica (71,927)** | Do Tasi (22,128) | Caleulema - 471 | Assorlema – 674 |
| | | Metagou – 1,677 | Lepa – 336 |
| | | Caimegoluli – 258 | |
| **Dili (277,279)** | Hera (62,848) | Erhetu(Aileu) – 262 | Sidole(Aileu) - 382 |
| | | Aidakbehare – 1,451 | Lebutu(Aileu) - 274 |
| **Manatuto (46,619)** | Komando (14,392) | Bamatac – 703 | Carlilu – 217 |
| | | Diric Un – 79 | |
| **Covalima (65,301)** | We Inan-Hasan (25,815) | Niquiir – 140 | Besac Oan – 720 |
| | | Ogues – 1,917 | Coloama – 697 |

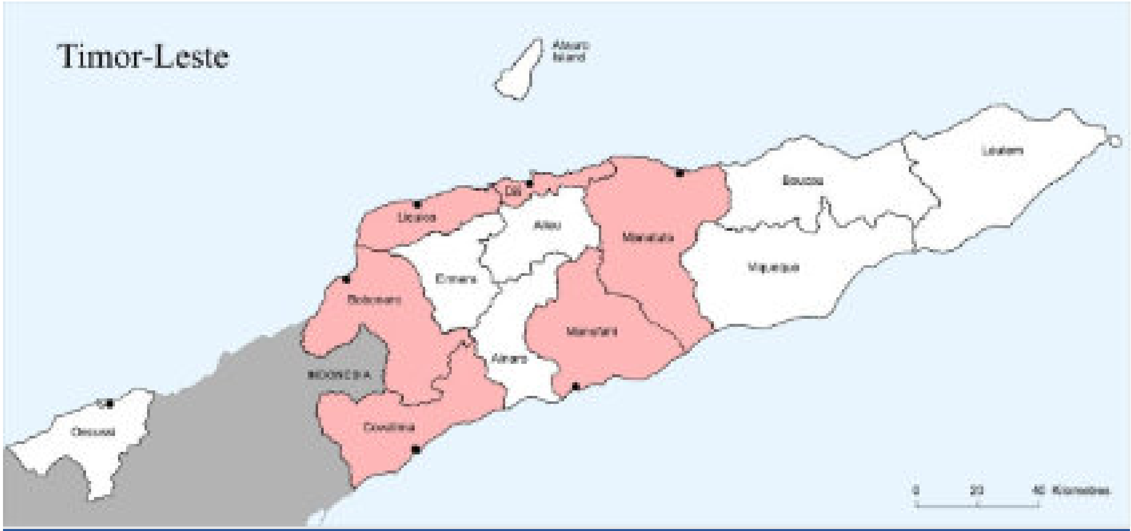


**Figure 2.** *Map of Timor-Leste showing municipalities. Red shading shows the municipalities represented by coastal producer sites.*

[1] Note: population number is to the level of *posto administrativo*. Data were unavailable to the level of aldeia (source: Direcção Geral de Estatística, Census 2015).

Coastal villages partaking in the FAD treatment are fishing villages with an active fleet of vessels. Baseline surveys at these sites will be fisheries focused questions aimed at establishing catch rates, income and relative wealth of fisher families prior to FAD deployment.
The SBCC level study population will be VSLA members and members of their households in inland villages with a particular focus on pregnant and lactating mothers, and children under 5 years of age.

29. Methods

Description of: (a) Population and sample, (b) Sampling techniques, sample size, inclusion and exclusion criteria, (c) Materials and procedures (if necessary), (d) Data management and analysis

Sampling, data types and collection procedures:

*Fisheriesproduction monitoring*

Fisheries catch monitoring methodology will be consistent with the national fisheries monitoring system in Timor-Leste, PeskAAS (as detailed in Tilley & Wilkinson 2020). This is a tablet-based survey that uploads near-real-time landings data to the central PeskAAS database, and is subsequently analyzed, aggregated and displayed automatically on an open access dashboard. Information collected includes the number of fishers (sex-disaggregated), boat and gear types, individual fish sizes and quantities of fish captured, trip duration, the primary habitat fished, and the proportion and price of fish sold. These data are also paired with geospatial vessel tracks for boats that are fitted with a GPS tracker. Fishing activities and catch monitoring will be established prior to installation of any FADs, to evaluate any change in catch rates or production volume following the FAD deployment. Enumerators at each of the four coastal sites will be responsible for daily data collection at landing sites of small-scale fishers, and will collect data on fish catch by family group.

Household surveys

Following enrolment and informed consent, a baseline survey will be conducted with one member per household. The baseline survey will consist of household demographic information, asset ownership, and food consumption questions to assess relative wealth and standard of living, access to fish and other animal source foods, food insecurity, and the frequency and volume of fish consumed. Qualitative 24-hour recalls will be done with the person responsible for preparing food for the household. If this person is not the caregiver for any children under five years old. The 24-hour recalls will be used to calculate the minimum dietary diversity for both the woman and child (FAO, 2010; World Health Organization, 2014). We will also ascertain if there are any cultural or traditional beliefs that influence fish consumption for specific people or at certain times.

Interviews will be conducted by research assistants extensively trained on the tools, methods, research ethics and confidentiality. Questionnaires will be translated into Tetum and carried out in Tetum or a combination of Tetum and other local languages. After enumerator training and survey pre-testing, the survey tools and procedures will be adapted as necessary to account for errors in interpretation by enumerators and respondents. Staff will inform the VSLA members in advance of the baseline and end line data collection sessions, and the latter will arrange for participants to be present.

All data will be kept confidential. Data will be submitted via 3G and only stored on password protected mobile phone tablets and laptops temporarily. Participants will be assigned randomly-generated ID numbers and names and identifying information will be de-linked from the dataset. Data will be analyzed for groups and no individual response will be identifiable. Survey data will be archived and accessible to only the research team and those approved by the entire research team. Datasets will be securely maintained indefinitely to facilitate future research for example pooled analysis with other studies for purposes of meta-analysis or multi-site analysis.

| | | | Week | | | | | | | | |
|---|---|---|---|---|---|---|---|---|---|---|---|
| Municipality | Village | N | 1 | 2 | 3 | 4 | 5 | 6 | 7 | 8 | 9 |
| Atabae, Bobonaru | Lia Bote | 30 | | | | | | | | | |
| | Rairobo | 30 | | | | | | | | | |
| | Lebulugor | 30 | | | | | | | | | |
| | Fatubesi | 30 | | | | | | | | | |
| | Irlelo | 30 | | | | | | | | | |
| Betano, Manufahi | Nonobuco | 30 | | | | | | | | | |
| | Loti | 30 | | | | | | | | | |
| | Raemeralau | 30 | | | | | | | | | |
| Do Tasi, Liquica | Caimegoluli | 30 | | | | | | | | | |
| | Metagoa | 30 | | | | | | | | | |
| | CaileoLema | 30 | | | | | | | | | |
| | Asorlema | 30 | | | | | | | | | |
| | Lepa | 30 | | | | | | | | | |
| Hera, Dili | Lebutu (Aileu) | 30 | | | | | | | | | |
| | Erhetu (Aileu) | 30 | | | | | | | | | |
| | Aidak behare | 30 | | | | | | | | | |
| | Sidole (Aileu) | 30 | | | | | | | | | |
| Komando, Manatuto | Diric-um | 30 | | | | | | | | | |
| | Bamatak | 30 | | | | | | | | | |
| | Carlilo | 30 | | | | | | | | | |
| We Inan-Hasan, Covalima | Ogues | 30 | | | | | | | | | |
| | Niquir | 30 | | | | | | | | | |
| | Coloama | 30 | | | | | | | | | |
| | Besan Oan | 30 | | | | | | | | | |
| 6 | 24 | 720 | 80 | 80 | 80 | 80 | 80 | 80 | 80 | 80 | 80 |

Sampling, data types and collection procedures: Data collection protocols per study objective

Initial visits to coastal sites will be used to conduct interviews and focus group discussions with fish traders who distribute and sell fish produced from each of these sites. A list of all nearby consumer sites (communities/markets) will be constructed from these interviews, and then these sites will be randomly assigned to treatment and control groups for the trial.

## Statistical analysis

### Main outcome variables

The main outcomes of interest are:

1) *Per capita household fish consumption* measured as total quantity (g) of fish consumed by the household in the previous seven days divided by the number adult equivalents in the household

2) *Frequency of fish consumed at household level* measured as the number of days fish were consumed out of the previous seven days.

3) *Frequency of fish consumption in women* measured as the number of days fish were consumed out of the previous seven days.

4) *Frequency of fish consumption in children* measured as the number of days fish were consumed out of the previous seven days.

**Other outcome variables:**

5) *Average catch rates* calculated as the increase in fish availability by comparing average catch rates prior to FAD deployment with catch rates post-deployment. Catch rate and total production values for different coastal community sites will be calculated and extrapolated following Tilley et al., (2020).

6) *Average price and supply of fish* calculated as the change in price and volume of fish available in upland village sites by comparing price and volume of fish proffered by traders at baseline with volumes at mid line and end line.

7) *Knowledge and practices about fish purchase and handling.* A knowledge test focusing on nutrition benefits of fish will be administered to all study respondents.

**Treatment variable**

The main treatment variable is a dummy variable that indicates to which of the four treatment arms a village was assigned. Our primary model is an unadjusted model.

30. Result expected (What results are expected)

This research will provide a baseline understanding of fish production at coastal sites and fish consumption in inland communities of 6 coastal districts of Timor-Leste.
It will provide results on the effect on catch rates and fisher incomes of installing fish aggregating devices at coastal sites.
It will provide results on the effect on fish consumption of a messaging (SBCC) campaign to members of VSLAs and other individuals in upland communities.
These two avenues of research will produce results of testing these two approaches as interventions to drive improved fish consumption by needy communities in Timor-Leste, and thereby provide valuable guidance and prioritization for GOTL and other stakeholders on scaling investment in these interventions as part of national programming.

31. Time table

| Activity | | | | | | Q1 2021 | Q2 2021 | Q3 2021 | Q4 2021 | | | | |
|---|---|---|---|---|---|---|---|---|---|---|---|---|---|
| Study design | | | | | | | | | | | | | |
| Approvals | | | | | | | | | | | | | |
| Enumeratorhiring and training | | | | | | | | | | | | | |
| Fisheriesmonitoring | | | | | | | | | | | | | |
| Surveypiloting | | | | | | | | | | | | | |
| Baseline survey | | | | | | | | | | | | | |
| Baseline analysis | | | | | | | | | | | | | |
| FADinstallation | | | | | | | | | | | | | |
| SBCCtool development | | | | | | | | | | | | | |
| SBCCtreatment | | | | | | | | | | | | | |
| Endline survey | | | | | | | | | | | | | |
| Analysisand write up | | | | | | | | | | | | | |

32. Funding (Breakdown of costs)

USD 150,000 Mercy Corps – development and deployment of SBCC messaging in 12 upland sites
USD 434,000 WorldFish – FADs (purchase, construction, and deployment), Project management, experimental design, survey development and enumerator training, reporting.

33. Signed by Principal Investigator:

| | |
|---|---|
| Signature | |
| Name | Alex Tilley PhD. |
| Date | 14 September 2020 |

PART C. SIGNATURES AND DECLARATION

- I certify that the information given is correct to the best of my knowledge.
- I acknowledge that I must notify the Committee in advance of any ethically-relevant variation to the project.
- I have read and agree to abide by the relevant parts of the Ethical and Technical Committee We promise to deposit the results of research and presented results to the CHRD

| NO | POSITION | NAME | SIGNATURE | DATE |
|---|---|---|---|---|
| 1 | Principal Investigator | Alexander Tilley | | |
| 2 | Co-Investigator 1 | Dedisio Jose Lay Ximenes | | |
| 3 | Co-Investigator 2 | Mario Pereira | | |

- I certify that I am aware of this project and its ethical issues.
- I agree that the Organization /Faculty will accept responsibility for the ethical conduct of the project as outlined above.

Organizational Head

| NO | POSITION | NAME | SIGNATURE | DATE |
|---|---|---|---|---|
| 1 | Director General, WorldFish | Gareth Johnstone Ph.D. | | |

PART D. Depart. Health Research MINISTRY OF HEALTH TIMOR-LESTE APPLICATION CHECKLIST

| No | Requirement | | Yes | No |
|---|---|---|---|---|
| 1. | Other Human Research Ethics Committee Consideration | Required? (*if so*, provide details under 'The Project' and attach copies of any responses already received) | | |
| 2. | Research Project Proposal | | | |
| 3. | Instruments (Questionnaires) | Required? (*if* so, attach to each copy of the application) | | |
| 4. | Consent Form | Required? (*if so*, attach to each copy of the application) | | |
| 5. | Resource | Required? (*if so*, attach any letters of support to each copy of the application) | | |
| 6. | Signatures | All Investigators | | |
| | | Principal Investigator's Supervisor | | |
| | | Organizational Head printed name and role in the Organization. | | |
| 7. | Time table | | | |
| 8. | Budget (Breakdown cost) | | | |
