# Supplementary material for: A supply and demand intervention increased fish consumption among rural women: A randomized, controlled trial
Source: PLoS One. 2026 Feb 19;21(2):e0340861. doi: 10.1371/journal.pone.0340861 (PMC12919792; doi:10.1371/journal.pone.0340861)
Supplement: Attachment — Submitted filename: Responses to reviewers.docx [file pone.0340861.s008.docx]

Responses to reviewers’ and editor’s comments.

| **Comment** | **Response** |
| --- | --- |
| **Editorial Request 1:** Ensure manuscript meets PLOS ONE style and file naming requirements. | Manuscript and associated files have been revised to fully comply with PLOS ONE formatting and file naming guidelines, following the provided templates. |
| **Editorial Request 2:** Explain changes to author list and confirm all authors meet authorship criteria. | We confirm all listed authors meet PLOS ONE authorship criteria. Changes reflect new contributors to data collection and analysis and manuscript drafting. Omissions of authors present in the protocol paper are because they left prior to data collection and were not part of the implementation of the study. |
| **Editorial Request 3:** Expand "CGIAR" acronym in financial disclosure. | "CGIAR" has been expanded to "Consultative Group on International Agricultural Research (CGIAR) Trust Fund" in the financial disclosure section. |
| **Editorial Request 4:** Remove funding information from Acknowledgments. | All funding-related information has been removed from the Acknowledgments and is presented only in the Funding Statement, as per journal requirements. |
| **Editorial Request 5:** Clarify data availability plan. | All anonymized data have been made available at WorldFish's Harvard Dataverse (<https://dataverse.harvard.edu/dataverse/worldfish>) and the Data Availability Statement has been updated accordingly. |
| **Editorial Request 6:** Address potential copyright issues with maps in Figure 1. | Figure 1 is compliant with CC BY 4.0. Figure legend has been updated accordingly. |
| **Editorial Request 7:** Move supplementary tables to Supporting Information files. | All supplementary tables have been moved to a Supporting Information file. |
| **Additional Editor Comment:** Specify RCT design, randomization, allocation, and blinding. | The study is a c**luster-randomized, 2×2 factorial, parallel-arm controlled trial.** Randomization was by Excel-generated random numbers; allocation was concealed at village level. Blinding was maintained for statistical analyses as far as feasible. This has been added to the start of section 2.2 |
|  |  |
| Reviewer 1 - The investigators gave much thought to the design of this study as is usually the case when a within cluster randomized approach is attempted. There were 4 interventions including and a control. The effect size was set at 15% with a 0.05 intra cluster correlation. The power was set at 0,80 and presumably a 0.05 alpha level. Provide some rationale for the intra cluster correlation value. | A footnote has been added to the text on correlation coefficients in the methods section.  “*The intra-cluster correlation coefficient (ICC) of 0.05 used in our sample size calculations is consistent with empirical ICC values reported in nutrition-sensitive agricultural and health behaviour cluster trials conducted in low- and middle-income countries. Prior studies in similar community-based interventions—particularly those measuring dietary behaviour outcomes—have used ICC estimates ranging between 0.01 and 0.10, with 0.05 being a commonly adopted mid-range conservative value to account for moderate within-cluster correlation (Kadiyala et al., 2021; Prost et al., 2013; Hayes & Moulton, 2009). Given that our primary outcomes involved household-level dietary behaviours influenced by shared environmental and cultural contexts within villages, we considered an ICC of 0.05 to be a reasonable and cautious estimate for power calculation.”* |
| **Reviewer 1 -** The primary outcome variable, the amount of fish purchased per household member over the past week (in grams), was log-transformed to achieve normality. Was this the only non-normal concern and how was it tested for normality? | This was the only non-normal concern emerging. Visual inspection of histograms followed by Shapiro-Wilk tests were used to assess normality before log-transformation. This is now described in the Methods section. |
| To assess the impact of Fish Aggregating Devices (FADs) on fish catch per unit effort (CPUE), their study implemented a difference-in-difference (DiD) methodology. This method analyses the variation in CPUE before and after FAD installation at the treatment sites and compares these to the changes at a control site where FADs were not deployed. GLM was used for continuous data and the logistic for categorical. Tables 5 and 6 give the primary outcomes for purchase patterns and fish consumption as well as the analysis approach for the specific endpoint. The overall conclusions appear to follow from the analyses performed. However, as per the protocol, any multiple comparisons adjustments were not obvious. This should be discussed in the manuscript text. | We did not apply a formal multiple-comparison adjustment because we only had two pre-specified primary outcomes (household fish purchases and women’s consumption) and treated each at α=0.05. All other tests were secondary/exploratory. This has been clarified in the methods text. |
| Reviewer 1 - There was limited info on confounder influence. Apparently more seasonal or environmental variables were referenced and those variables were included as confounders in the impact evaluation models. If confounders remained significant in the full model (at p<0.05), they were retained. The actual role or significance of expected confounders were not detailed statistically in the logistic or GLM outcomes. The discussion of data management was present, but not detailed in terms of quality control which may not have been the intent in this report. Likewise, the same concern about the detailed discussion for the spillover and mediation analysis which were performed. These concerns should be addressed. | Confounders were identified by bivariate analysis (p<0.10) and retained in final models if significant. Methods and Results sections now describe this process clearly. |
| Some important limitations were noted by the investigators such as possible response bias on over consumption and food sharing under reporting. Were any steps taken to validate these responses? | While no external validation was conducted, standardized training and quality control procedures were employed to minimize response bias. This is now clarified in the text. We have also added some text to the discussion of limitations specifically about reporting bias and how we consider this (section 4.2). |
|  |  |
| **Reviewer 2 -** The title needs modification Like this effect of ……….. | *Title has been revised to: " A supply and demand intervention increased fish consumption among rural women: a randomized, controlled trial"* |
| Reviewer 2 - Why are you targeting women? | Although fish purchasing was surveyed at the household level, women were targeted with 24hr recall because of their central role in household food dietary decisions (especially for their children) in rural Timor-Leste.  By increasing awareness and knowledge of better diets with women, the hypothesis is that this will drive improved child nutrition directly and indirectly through better household diets. This rationale is now included in the Introduction.  *“Increasing women’s dietary intake and nutrition knowledge has been shown to improve overall household food choices and is strongly associated with better child health and nutrition outcomes, particularly in the critical first 1,000 days [19].*” |
| **Reviewer 2 -** What is the novelty of your study since many studies done with similar topics in the country? | We contest that many studies with similar topics have been conducted in Timor-Leste. Much research conducted in Timor is focused on nutrition, but this seems justified given it is one of the most malnourished nations in the world, with nearly 50% of children stunted.  To the best of our knowledge, this is the first study of its kind to use an RCT to assess a nutrition-sensitive fisheries intervention in the world. This is highlighted in the Introduction and Discussion. |
| **Reviewer 2 - Abstract:**  The background - it would be better if you focused on the severity or magnitudes of the problems or the Gap. Results: is not written clearly, so would you re-write again Conclusion: would you write it based on your findings What is the clinical implication of your findings? Does the trial registered? where? pls would you include the registration number? | *We have reworked the abstract based on your comments and included the clinicaltrials.gov registration number, as reported in the registered protocol.* |
| **Reviewer 2 - Trial registration:** Include registration details. | The details of the trial registration are stated at the end of the methods (# NCT04729829) (line 243 of the original ms). We have added this to before the abstract also. |
| **Reviewer 2 - Introduction:**  Para 1,2 and 3 are redundant as they have no direct relevance to the current objectives of the study, and are not required for the audience of the Journal of PLOS ONE.  The introduction section is too short and not clear, and it still is unable to appropriately justify neither the need for this study nor the target age group. Some of the information provided is excessive and not necessary as the reader quickly becomes inundated with an overabundance of information. Incidentally, the authors should try to provide a more succinct background by truncating/condensing some of the information to provide more specific In general, following the idea is difficult to understand.  It would be better if you started from definition ---- magnitudes ------ impact ------- etc What was your hypothesis ? try to include it | We have substantially restructured the intro in response to your comments and included a specific hypotheses section following the published protocol. We propose retaining Paragraph 1 as it serves an important framing function. This paragraph positions fish within the broader discourse of food systems transformation in low- and middle-income countries (LMICs), where persistent malnutrition and environmental degradation challenge conventional agricultural models. Specifically, it highlights that fish and other aquatic foods are increasingly recognized as underutilized but highly efficient sources of micronutrients, with relatively low environmental footprints compared to terrestrial animal-source foods. These attributes are highly relevant to LMIC contexts like Timor-Leste, where nutritional deficiencies are widespread and sustainable food system interventions are urgently needed. Recent literature supports this framing, showing that fish provide bioavailable nutrients essential for maternal and child health, particularly in the first 1,000 days, and that small pelagic fish can play a pivotal role in achieving dietary adequacy in resource-limited settings (e.g., Robinson et al. 2022 [8]; Byrd et al. 2021 [3]).  We believe this framing helps to establish the relevance and global applicability of the study and clarifies why a fisheries-based intervention warrants rigorous testing in the context of nutrition-sensitive food systems. However, we have reviewed and tightened the subsequent paragraphs for conciseness and clarity in response to the reviewer’s broader concerns. |
| **Reviewer 2 - Methods:**   - Make it clear what is your sampling frame or unit? - How did you use a simple sampling technique to select women? - Do you have references for the operational definition? - How did you control information contamination? - Why didn’t conducted t-test analysis - Have you done difference in difference analysis? If not why? - Did you conduct GEE analysis? If not why - How did you check the normality distribution at baseline and endline? - Did you calculate the effect size and interaction effect? | We have clarified the sampling technique, and that the sampling unit was the household in inland villages, and the respondent within the household was the primary food purchaser, typically an adult woman. (section 2.6.2)  Added: Dietary diversity was assessed using a qualitative 24-hour recall following FAO’s Minimum Dietary Diversity for Women guidelines (Section 2.6.3)  Contamination Control: Mapping of trader routes and purposive village selection minimized contamination between intervention and control sites as stated in section 2.2  We used general linear models (for continuous outcomes) and logistic regression (for binary outcomes) rather than t-tests, as these allowed for the adjustment of clustering and covariates. These methods are more robust for the design and data structure of our cluster-randomized trial. (2.7)  We conducted a difference-in-difference analysis to evaluate the effect of FADs on CPUE at coastal landing sites, and this is stated in the statistical methods section (2.7)  We used mixed effects generalized linear models with robust standard errors to account for clustering at the village level. We opted for this approach over GEE due to our relatively small number of clusters, where mixed models with robust errors are preferred for unbiased estimation. (2.7)  Description of normality assessment has been added in response to comments from Reviewer 1 also. (2.7) |
| **Reviewer 2 – Check the results tablets once again** | All tables have been reviewed again |
| Reviewer 2 - Discussion:   - Need major revision - The discussion needs to be focused on the main objectives. - The functional significance of the present results may be elaborated. - Please include the strengths and limitations of the study. - What is the clinical implication of your findings? - Pls include the clinical implication and future direction of your findings. | The discussion has been rewritten to focus on the main objectives and outcomes and now includes sections on 4.1 clinical and public health implications, 4.2 strengths and limitations 4.3 policy and systems implications, and 4.4 future directions. |
| **Reviewer 2 - Ethical considerations:** Include approval number and date. | Ethical approval number and date (1934MS-INS/DE/XII/2020) have been added to the Ethics section. |
| **Reviewer 2 - References:** Correct format. | References have been reviewed and reformatted to comply with PLOS ONE style guidelines. |
