# Supplementary material for: A supply and demand intervention increased fish consumption among rural women: A randomized, controlled trial
Source: PLoS One. 2026 Feb 19;21(2):e0340861. doi: 10.1371/journal.pone.0340861 (PMC12919792; doi:10.1371/journal.pone.0340861)
Supplement: Attachment — Submitted filename: Response to reviewers.docx [file pone.0340861.s010.docx]

We thank the reviewer for their thoughtful review and helpful comments that have strengthened the clarity and rigor of the manuscript. Below, we provide point-by-point responses to each of the issues raised.

**General Comments**

| **Reviewer Comment** | **Response** |
| --- | --- |
| The methodology description is difficult to read and can probably be streamlined. The explanation of the analysis is unnecessarily complicated (e.g. L355ff). | We have revised the Methods section to streamline the explanation of statistical analyses. The description of DiD, GLM models, and bootstrap methods has been simplified for better readability while retaining technical accuracy. |
| it is mentioned repeatedly that FADs are cost-effective and affordable, but given that the FADs in two regions suffered from lower catches than in the reference period without them, this is not quite obvious, especially if fishers would be expected to pay for the installation themselves (presumably, the cost was borne by the project during the testing). | We have clarified in the Discussion that while FADs can be cost-effective under favourable ecological and social conditions (as seen in Bobonaro), but also that this is not universally true. The project covered the costs in this trial, and we have explicitly noted that cost-effectiveness may not hold where FADs fail to increase catch. |
| Only towards the end is there talk about the perishable nature of (fresh) small pelagics marketed inland within 30 km from the landing sites. However, a case of a mother adding fish powder to the food of her infant suggests a dried form of marketing. | We agree and have added to the discussion to highlight earlier the emerging research and implementation of dried fish and fish powder in Timor-Leste, and emphasize that access to fish in inland upland areas is expanding to include processed fish products with greater shelf life. |
| Perhaps more than the great potential to address nutritional deficits the pilot shows the challenges of changing food habits under the constrained conditions of the rural population that requires considerable multi-pronged approaches to have a chance of success at scale. This obviously should not discourage efforts at ensuring nutritious food, particularly for infants and women in child-bearing age, but may also invite a horizon scan for other options to achieve that objective. | We appreciate this insight and have revised the final paragraph of the Discussion to reflect that while promising, the intervention illustrates the complex, multi-pronged strategies required to improve nutrition sustainably. |

**Specific Comments**

| **Reviewer Comment** | **Response** |
| --- | --- |
| **Line 190**: correct kg/fisher/hr to kg/fisher × hr | Corrected as suggested. |
| **Footnote on page 11**: citations use names, not numbers. References are missing from bibliography. | We have corrected the citation style to numbered references and ensured that all cited sources are included in the reference list. |
| **Line 269**: SBCC | “SBCC” has been replaced with the consistent term “SBC” throughout the manuscript. |
| **Line 262 and 275**: STATA 13 vs STATA 14 | We have corrected line 262 to state that we used Stata 14. Stata 13 was an error. |
| **Line 352**: Can this be expressed positively? Does this imply SBC made no difference in women’s knowledge? | We have revised the sentence to clarify that knowledge of fish benefits was already very high at baseline, leaving limited room for measurable improvement. This does not imply SBC had no effects on fish consumption in general, but rather that knowledge of fish consumption benefits were already high so would not change between baseline and endline. |
| **Line 358**: “FAD and SBC only arm” | Corrected to “FAD + SBC arm” for clarity and consistency. |
| Line 364f: reference is made to Table S2 and S3: Women are more than 8 times as likely to consume fish? But in Line 395 there is talk of them being 4 times more likely. Evidence needs to be shown and harmonised and the descriptor in Table S2 corrected to: Mean quantity in grams (CI) of fish purchase/household member x weekb. | The 8x estimate comes from the exploratory analysis where the “treatment” arm was redefined based on actual CPUE increases (i.e. sites with FADs that did not show an increase in catch rate were recoded to ‘control’) . The earlier reported result of “4x as likely to consume fish” comes from the primary analysis (reported in the protocol).  Table S2 and S3 also refer to the exploratory analyses. The effect size of women reporting fish the day before in the primary analysis had a PR of 4.18 (Table 6), while in the exploratory analysis we found a PR of 8.5 (Table S3). We have added these relevant statistics to the manuscript text also. |
| **Line 413**: correct grammar | Grammar corrected to “A comprehensive examination…”. |
| **References**: 60 cited in-text, only 47 in layout | Reference list has been reviewed and updated. All in-text citations now have a corresponding entry in the bibliography. |
| **Table S1**: Decapterus macarellus should be italicized | Corrected as suggested. |
